# Proteomic Differences between Developmental Stages of *Toxoplasma gondii* Revealed by iTRAQ-Based Quantitative Proteomics

**DOI:** 10.3389/fmicb.2017.00985

**Published:** 2017-06-02

**Authors:** Ze-Xiang Wang, Chun-Xue Zhou, Hany M. Elsheikha, Shuai He, Dong-Hui Zhou, Xing-Quan Zhu

**Affiliations:** ^1^State Key Laboratory of Veterinary Etiological Biology, Key Laboratory of Veterinary Parasitology of Gansu Province, Lanzhou Veterinary Research Institute, Chinese Academy of Agricultural SciencesLanzhou, China; ^2^National Animal Protozoa Laboratory and College of Veterinary Medicine, China Agricultural UniversityBeijing, China; ^3^Faculty of Medicine and Health Sciences, School of Veterinary Medicine and Science, University of NottinghamLoughborough, United Kingdom; ^4^College of Animal Science and Technology, Anhui Agricultural UniversityHefei, China; ^5^Jiangsu Co-innovation Center for Prevention and Control of Important Animal Infectious Diseases and ZoonosesYangzhou, China

**Keywords:** *Toxoplasma gondii*, life-cycle, mass spectrometry, proteomics, iTRAQ, differentially expressed protein (DEP)

## Abstract

*Toxoplasma gondii* has a complex two-host life-cycle between intermediate host and definitive host. Understanding proteomic variations across the life-cycle stages of *T. gondii* may improve the understanding of molecular adaption mechanism of *T. gondii* across life-cycle stages, and should have implications for the development of new treatment and prevention interventions against *T. gondii* infection. Here, we utilized LC–MS/MS coupled with iTRAQ labeling technology to identify differentially expressed proteins (DEPs) specific to tachyzoite (T), bradyzoites-containing cyst (C) and sporulated oocyst (O) stages of the cyst-forming *T. gondii* Prugniuad (Pru) strain. A total of 6285 proteins were identified in the three developmental stages of *T. gondii*. Our analysis also revealed 875, 656, and 538 DEPs in O vs. T, T vs. C, and C vs. O, respectively. The up- and down-regulated proteins were analyzed by Gene Ontology enrichment, KEGG pathway and STRING analyses. Some virulence-related factors and ribosomal proteins exhibited distinct expression patterns across the life-cycle stages. The virulence factors expressed in sporulated oocysts and the number of up-regulated virulence factors in the cyst stage were about twice as many as in tachyzoites. Of the 79 ribosomal proteins identified in *T. gondii*, the number of up-regulated ribosomal proteins was 33 and 46 in sporulated oocysts and cysts, respectively, compared with tachyzoites. These results support the hypothesis that oocyst and cystic stages are able to adapt to adverse environmental conditions and selection pressures induced by the host's immune response, respectively. These findings have important implications for understanding of the developmental biology of *T. gondii*, which may facilitate the discovery of novel therapeutic targets to better control toxoplasmosis.

## Introduction

*Toxoplasma gondii* infection represents a significant global health burden, with considerable social and economic implications. This parasite can infect all vertebrate animals and humans (White et al., 2014). Although most immuno-competent individuals do not develop clinical disease, *T. gondii* infection in immuno-compromised individuals, such as AIDS or cancer patients, can cause severe encephalitis and retinochoroiditis (Suzuki et al., 1996; Kijlstra and Jongert, 2008; Herrmann et al., 2012). Also, congenital infection with *T. gondii* is a major cause of spontaneous abortion, preterm labor, or significant diseases in the survived neonate (Montoya and Liesenfeld, 2004). Current treatments are expensive and toxic (McLeod et al., 2006); and together with the lack of a vaccine makes the need for new therapeutic agents very urgent. Development of new treatment and prevention approaches including anti-*T. gondii* drugs and vaccines can be most potent if targeted at specific life-cycle stages and/or specific proteins expressed at these developmental stages. Therefore, better understanding of the developmental biology and stage-specific molecular determinants of the life-cycle stages is urgently needed.

*Toxoplasma gondii* has a complex, two-host life-cycle that involves both asexual (takes place in any vertebrate animal as an intermediate host) and sexual (occurs only in the felid definitive host) reproductive cycles (Blader et al., 2015). The sexual cycle of *T. gondii* occurs exclusively in the enteroepithelial cells of the intestine of the felid's host (Behnke et al., 2014) and culminates in the production of oocysts, which are adapted to survive under adverse climatic conditions (Dubey, 2010). *Toxoplasma gondii* oocysts are shed non-sporulated (non-infective) in cat feces, and in a few days they undergo a sporulation process in the environment and with appropriate climatic conditions they mature to sporulated oocysts. In the intermediate host, *T. gondii* undertakes asexual reproduction cycle, which follows ingestion of sporulated oocysts or ingestion of tissue containing *T. gondii* cysts full of bradyzoites (Dattoli et al., 2011). Asexual cycle involves two different tissue stages produced in different phases of the infection process and play different roles. These two stages exist as a replicative tachyzoite stage (Hehl et al., 2015), which can transform to slowly replicating bradyzoites within a tissue cyst, which are commonly found in the central nervous system (Bohne et al., 1999). Both of tachyzoite and bradyzoite are infective to cats.

Advances in proteomic technologies have already contributed to an increasing understanding of the protein expression in *T. gondii*. For example, in 2002 the first proteome map of *T. gondii* tachyzoite was constructed using 2-dimensional electrophoresis (2-DE) combined with matrix-assisted laser-desorption/ionization time of flight mass spectrometry (MALDI-TOF-MS) (Cohen et al., 2002). Zhou et al. employed two-dimensional difference gel electrophoresis (2D-DIGE) coupled with MALDI-TOF-MS to compare the differentially expressed proteins of four different genotypes of *T. gondii* tachyzoites (Zhou et al., 2014). Also, proteins expressed by oocysts and sporozoites of *T. gondii* have been studied (Fritz et al., 2012; Possenti et al., 2013). However, global protein expression patterns across the developmental stages of *T. gondii* have yet to be determined. In order to understand the functional differences among different stages of *T. gondii* life-cycle it is important to identify and quantify the protein's content of these stages.

Comparative proteomic analysis requires the use of exceptionally sensitive mass spectrometric approaches. In this study, we utilized LC-MS/MS analysis coupled with isobaric tags for relative and absolute quantification (iTRAQ) labeling and SCX fractionation to identify and quantify differentially expressed proteins of different stages of *T. gondii* life-cycle. iTRAQ is a quantitative proteomic method that can generate information on the abundance of hundreds of proteins at one time. Also, it allows parallel biological or technical replicates to be multiplexed (4-plex or 8-plex iTRAQ labeling) in one LC-MS/MS experiment, thus overcomes the inter-assay variations that occur in a single MS-based shotgun profiling experiment (Wu et al., 2006; Pierce et al., 2008; Craft et al., 2013). Also, this method has been successfully used to determine the differentially expressed proteins of *T. gondii* oocysts during sporulation (Zhou et al., 2016). The present study aimed to unravel the set of proteins that are unique to three life-cycle stages of *T. gondii*, namely oocyst, tachyzoite, and bradyzoites-containing cyst using 8-plex iTRAQ labeling and LC-MS/MS. We identified differentially expressed proteins, which are specific to the three infective life-cycle stages of *T. gondii*.

## Materials and methods

### Ethics approval

All protocols were reviewed and approved by the Animal Research Ethics Committee of Lanzhou Veterinary Research Institute, Chinese Academy of Agricultural Sciences. The experiments were performed in a strict accordance with the Animal Ethics Procedures and Guidelines of the People's Republic of China. All efforts were made to minimize suffering of the animals.

### Mice, cats, and parasite strain

Six to eight week-old female BALB/c mice and 10 week-old female kitten were obtained from Laboratory Animal Center of Lanzhou Veterinary Research Institute. Type II Prugniuad (PRU) strain of *T. gondii* was maintained in our laboratory by passaging the cysts in mice as described previously (Zhou et al., 2016).

### Preparation of parasite materials

#### Collection of tachyzoites

Tachyzoites of Type II Prugniuad (PRU) strain were collected using a previously described method (Zhou et al., 2011, 2014). Briefly, specific-pathogen free (SPF) mice were treated with 0.2 mg of dexamethasone (DSMS) every other day for three times and then inoculated orally with 100–150 cysts. Nine days after infection the peritoneal cavity was rinsed with sterile phosphate-buffered saline (PBS, 137 mM NaCl, 2.7 mM KCl, 10 mM Na_2_HPO_4_, 2 mM KH_2_PO_4_) to harvest the tachyzoites. Then, the peritoneal wash containing the tachyzoites was collected and centrifuged for 15 min at 1,680 *g* followed by two further washes with PBS. After the final wash the supernatant was decanted and the pellet was digested with 0.25% trypsin at 37°C for 20 min and centrifuged for 15 min at 1,680 *g*. The supernatant was removed and the final protein pellet was suspended in 1 mL PBS and was kept in an Eppendorf tube at −80°C until analysis.

#### Isolation and purification of cysts

Mice were infected orally with 100–150 *T. gondii* cysts and were humanely sacrificed 4 weeks later. Mice brains were collected and grinded with a pestle and mortar. During grinding, 1 ml of PBS per mouse brain was added slowly as grinding proceeds. The brain homogenate was layered on the top of Lymphocyte Separation Medium (Solarbio, Beijing, China) in a plastic tube followed by centrifugation at 2,290 *g* for 30 min. The parasite cysts were harvested and kept at −80°C until use.

#### Preparation of sporulated oocysts

Specific-pathogen-free kitten was infected with 200 cysts recovered from mice brain and its feces was collected daily to isolate oocysts using caesium chloride (CsCl) centrifugation method as described previously (Staggs et al., 2009). Briefly, fecal samples were mixed in water and filtered through 250 μm mesh. The filtrates were pelleted by centrifugation and the supernatant was discarded. Following three washes in PBS, the supernatant was discarded and the pellet was mixed with 5 volumes of sucrose solution with 1.15 specific gravity. The supernatant containing oocysts were collected, suspended in TE buffer (10 mM Tris-HCl, 1 mM EDTA, pH = 8.0) and centrifuged using discontinuous CsCl gradient method. Oocysts at the opaque-to-white layer interface were collected and washed with 0.85% saline. Finally, the oocyst pellet was suspended in PBS and maintained at 4°C. To induce sporulation, the oocyst's suspension was centrifuged and the pelleted oocysts were mixed with 2% H_2_SO_4_, and were maintained in an aerobic condition at ambient temperature for 7 days. Then the oocyst's suspension was washed with 0.85% saline, and the mature/sporulated oocysts were mixed with 2% H_2_SO_4_ and stored at 4°C until analysis.

### Protein extraction

Proteins were extracted from at least two biological repeats of each of the life-cycle stages, namely tachyzoites (~10^8^), cysts (~10^7^) and oocysts (~10^7^). Briefly, each biological sample was homogenized with 5 volumes glass sand and lysed in 200 μl radioimmunoprecipitation assay (RIPA) buffer (20 mM Tris-HCl pH 7.5, 150 mM sodium chloride, 2 mM EDTA, 1% DOC, 1% Triton X-100) containing phenylmethylsulfonyl fluoride (PMSF). Then, samples were sonicated (2%, 1 s ON and 1 s OFF cycle, 5 times, 8 repeats) and the supernatant containing total soluble proteins was collected after 20-min centrifugation at 13,400 *g*. The concentration of the protein was determined by the protein quantitative kit (QuantiProTM BCA Assay Kit, Sigma).

### Protein digestion and iTRAQ labeling

For protein digestion, 100 μg protein of each sample was reduced, alkylated, and then precipitated by the methanol/chloroform precipitation method. Firstly, 50 mM Reducing Reagent (final concentration, 8-plex iTRAQ kit, AB Sciex, USA) were added into each sample followed by incubation at 60°C for 1 hr. Then, the sample was mixed with 200 mM Cysteine-Blocking Reagent (final concentration, 8-plex iTRAQ kit, AB Sciex, USA) and held at ambient temperature for 10 min. Following rinsing the 10 KD ultrafiltration cartridge with 70% ethanol and deionized water, the protein solution was poured into ultrafiltration cartridge and centrifuged at 13,400 g for 20 min. Solution at the bottom of ultrafiltration cartridge was discarded and the ultrafiltration cartridge was centrifuged with 100 μl 0.25M TEAB (triethyl ammonium bicarbonate) three times at 13,400 *g* for 20 min. The protein pellets were reconstituted in 50 μl of 6 M urea/50 mM TEAB with sonication (Possenti et al., 2013) and digested in 2% trypsin overnight (Promega). Then, the digested peptides were dried and reconstituted in 0.5 M TEAB. Then, peptides were labeled according to the instructions of 8-plex iTRAQ kit (Applied Biosystems/MDS Sciex, Foster City, CA). Label reagent mixed with 150 μl isopropyl alcohol was mixed with samples and incubated for 2 hr at ambient temperature. The reaction was terminated with deionized water and samples were thoroughly mixed. Following vacuum centrifugation, dried samples were preserved at −80°C until use. Proteins of sporulated oocysts were labeled with 115 and 116, whereas the proteins of the cysts and tachyzoites were labeled with 117, 118, and 119, 121, respectively.

### SCX fraction and LC-MS/MS analysis

Strong cation exchange (SCX) fractionation chromatography was performed using the high performance liquid chromatography (HPLC) system (Phenomenex columns; Gemini-NX 3u C18 110A; 150^*^2.00 mM). The iTRAQ-labeled peptides were separated by a linear gradient formed by mobile phase A (20 mM HCOONH4, pH 10) and mobile phase B (20 mM HCOONH4, 80% ACN, pH 10). The flow of peptides elution was set to a constant rate of 200 μl/min. A total of 24 fractions were collected by a linear gradient (1 collection every 1 min, 100 min) and were acidified with trifluoroacetic acid (50%). The fractions were vacuum-dried for further analysis with LC-MS. Fractions (96 μg) were dissolved in buffer A [0.1% formic acid, 2% acetonitrile (ACN)] and pelleted at 13,447 *g* for 20 min. The supernatant was loaded onto analytical columns and was identified with an online Q Exactive system (Thermal Scientific). Component of mobile phase A and mobile phase B of LC-MS was formic acid (0.1%) and 80% ACN containing 0.1% formic acid, respectively. The flow rate of analytical columns was set at 350 nl/min and the peptides were analyzed within 65 min 3-step gradient (80% ACN in 0.1% formic acid from 4 to 50% over 45 min, 50 to 90% over 5 min and kept at 90% for 5 min). The parameters of the first grade MS included scan ranges from 350 to 1,800 m/z at a resolution of 70,000 with a maximum injection time of 40 ms. The second grade MS spectra were acquired in a resolution of 17,500 with 60 ms maximum injection time and the 20 top precursors for each MS cycle were selected.

### Database search and bioinformatics analysis

The raw MS data was transformed into mascot generic format (.mgf) files with Proteome Discoverer™ 1.4 and the data file was used to query *T. gondii* ME49 strain database (http://www.toxodb.org/common/downloads/release-10.0/TgondiiME49/fasta/data/), which contains 8322 protein sequences. The ProteinPilot™ Software 4.5 (AB SCIEX) was used to further identify and quantity proteins. To filter the results, we employed false discovery rate (FDR) of less than 0.01 for identification and the confidence level of 95% or unused confidence score larger than 1.3 for quantification. For differentially expressed proteins (DEPs), those with |log_2_ fold change|>1 were deemed upregulated or downregulated proteins, respectively.

Functional classification of the DEPs was performed according to Gene Ontology (GO) annotation and enrichment (http://www.geneontology.org) analysis. The DEPs were classified into three categories, namely molecular function, biological process and cellular component. The Kyoto Encyclopedia of Genes and Genomes (KEGG) (http://www.kegg.jp/kegg/) was used to predict molecular function, biological processes and significant DEPs pathways. Protein-protein interactions (PPI) were predicted in the Search Tool of the Retrieval of Interaction Genes/Proteins (STRING) database and the interaction network was illustrated by Cytoscape software. The PPI whose combined score was >0.9 were subjected to further interaction network analysis.

## Results

### Overview of primary data and protein identification

**Figure 1:**
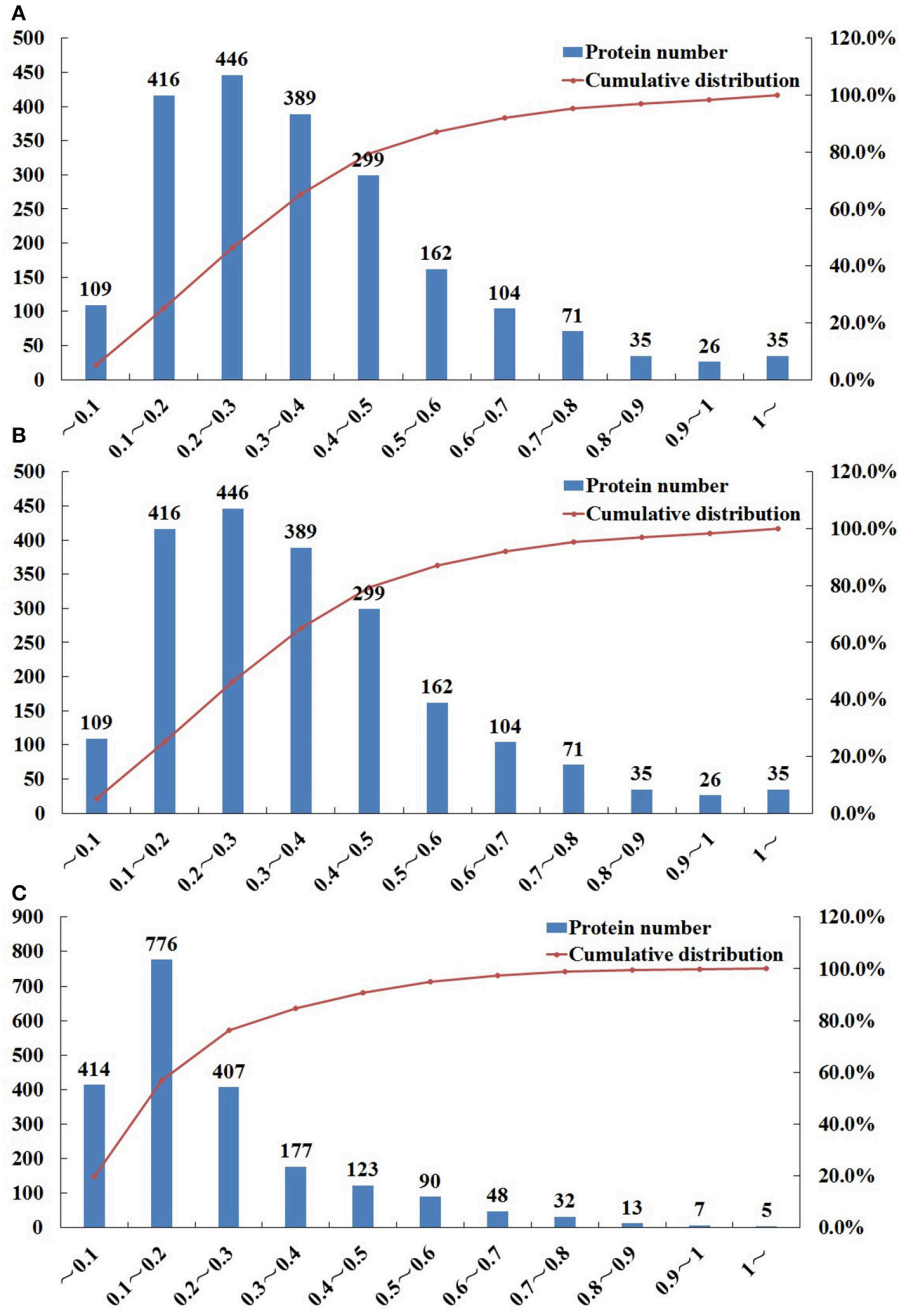
Repeatability analysis based on Coefficient of Variation (CV) of O vs. T, T vs. C and C vs. O. The x-axis represents values of CV. The y-axis on the left represents the number of proteins. The y-axis on the right represents the cumulative percentage of proteins. **(A–C)** represent repeatability analysis of O vs. T, T vs. C and C vs. O, respectively. O, oocysts; T, tachyzoites; C, bradyzoites-containing cysts.

iTRAQ was used to identify the proteomic differences among life-cycle stages of *T. gondii*, namely sporulated oocyst, tachyzoite, bradyzoites-containing cyst. Representative SCX chromatogram of the results is shown in Figure S1. A total of 6,285 proteins were identified from 53,335 peptides, which were matched with 76,534 spectra at a false discovery rate of 1%. Multiple proteins were identified, among which, 1667, 1697, and 1791, proteins were identified in O vs. T (oocysts vs. tachyzoites), T vs. C (tachyzoites vs. cysts) and C vs. O (cysts vs. oocysts), respectively after filtration (CV ≤ 0.5) (Tables S1–S3). Using 99% confidence level (CV≤0.5), 1602, 1625, and 1719 proteins were identified in O vs. T, T vs. C, and C vs. O, respectively (Tables S4–S6). Compared with analysis using 95% confidence level, 65 proteins, 72 proteins and 72 proteins were lost in O vs. T, T vs. C, and C vs. O. Repeatability analysis based on the Coefficient of variation (CV) showed that 79.3, 81.8, and 90.7% proteins can be covered in the total identified proteins in O vs. T, T vs. C, and C vs. O, respectively, when the CV was less than 0.5 (Figure 1).

### Protein quantification and hierarchical clustering analysis

**Figure 2:**
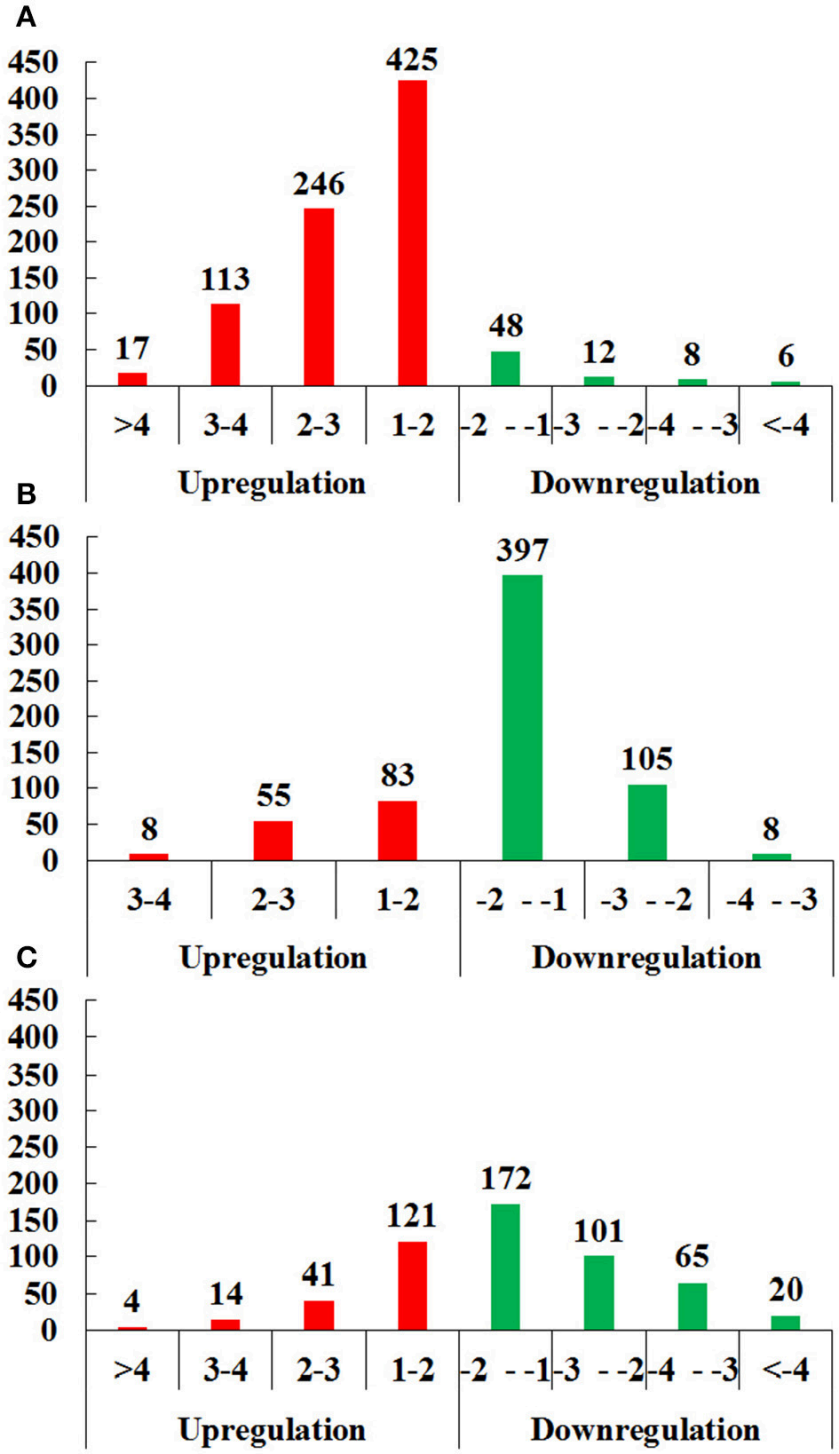
Distributions of differentially expressed proteins (DEPs) of O vs. T, T vs. C and C vs. O in different level of differentiation. **(A–C)** represent distributions of DEPs of O vs. T, T vs. C and C vs. O, respectively. The x-axis indicates values of log_2_ fold changes. The y-axis indicates the number of proteins. Red and green colors represent upregulated and downregulated DEPs. O, oocysts; T, tachyzoites; C, bradyzoites-containing cysts.

**Figure 3:**
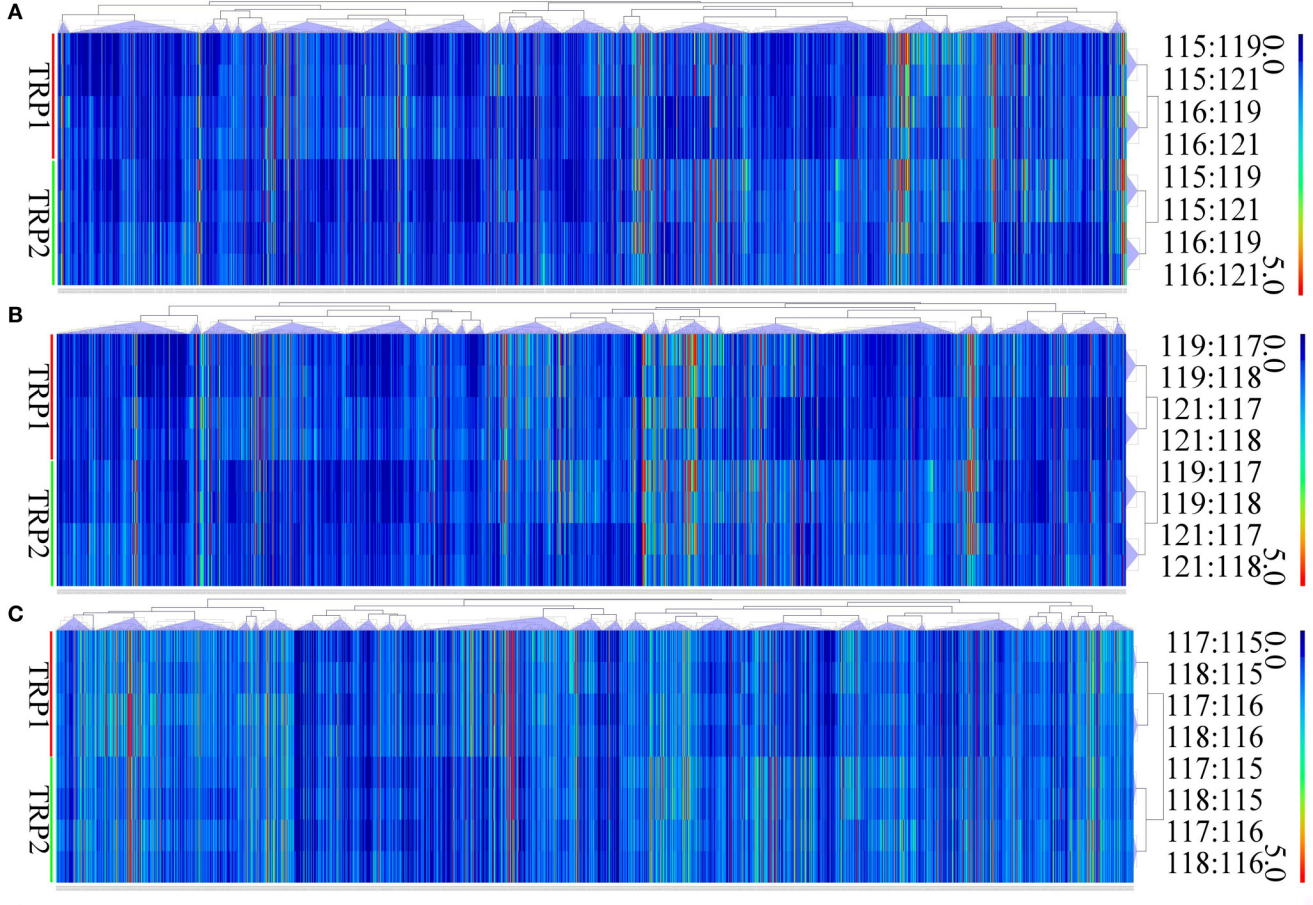
Hierarchical clustering of DEPs of O vs. T, T vs. C and C vs. O. Expression values were log_2_-transformed. TRP1 and TRP2 represent biological replicates 1 and 2. **(A–C)** represent hierarchical clustering of DEPs for O vs. T, T vs. C and C vs. O, respectively. O, oocysts; T, tachyzoites; C, bradyzoites-containing cysts.

In brief, 875 proteins, 656 proteins, and 538 proteins were defined as differentially expressed protein (DEPs) in O vs. T, T vs. C and C vs. O, respectively (|log_2_ fold change|>1 and *P* < 0.05) (Tables S7–S9). Among DEPs in each group, 801 proteins and 74 proteins were upregulated or downregulated in oocyst compared with tachyzoite stage. There were 146 upregulated proteins and 510 downregulated proteins in T vs. C. Further, 180 proteins and 358 proteins had higher or lower expression level in cyst than in oocyst. Numbers of DEPs in different level are presented in Figure 2. Results of the hierarchical clustering analysis of the identified proteins are shown in Figure 3.

### Gene ontology analysis of DEPs

**Figure 4:**
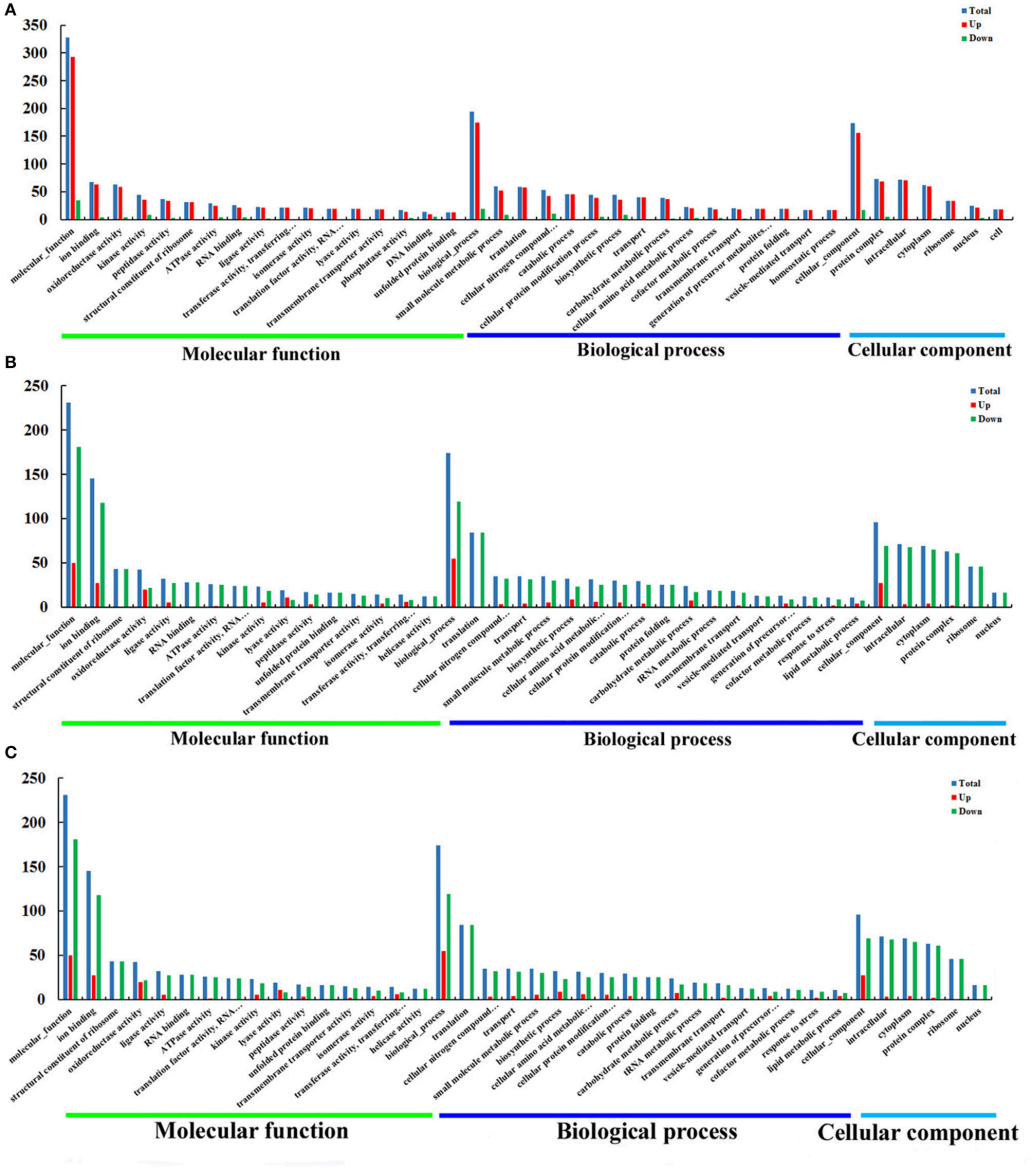
GO enrichment analysis of DEPs of O vs. T, T vs. C and C vs. O. DEPs in each group were sorted into three categories. The x-axis means different GO terms. The y-axis represents the number of proteins in the indicated categories. **(A–C)** represent GO enrichment analysis of DEPs for O vs. T, T vs. C and C vs. O, respectively. O, oocysts; T, tachyzoites; C, bradyzoites-containing cysts.

The DEPs were subjected to functional classification by Gene Ontology (GO) analysis. We identified 3371, 2846, and 1998 GO terms in O vs. T, T vs. C and C vs. O, respectively. Among these GO terms, there were 1708 molecular function terms, 874 biological process terms and 789 cellular component terms in O vs. T. GO terms in T vs. C included 1392 molecular function terms, 832 biological process terms, 622 cellular component terms and GO terms in C vs. O included 1083 molecular function terms, 634 biological process terms and 281 cellular component terms. Those GO terms were classified and enriched in order to investigate the properties of upregulated and downregulated proteins in each group. The most 40 enriched GO terms in each group and their destination to three main GO categories are presented in Figure 4.

As is shown in Figure 4A, for O vs. T, the top five enriched GO terms for upregulated proteins within molecular function were ion binding, oxidoreductase activity, kinase activity, peptidase activity and structural constituent of ribosome. The top five enriched GO terms for downregulated proteins were kinase activity, DNA binding, helicase activity, RNA binding, and oxidoreductase activity. Translation, small molecule metabolic process, catabolic process, cellular nitrogen compound metabolic process and transport were the five top terms for upregulated proteins under biological process. The top five enriched GO terms for downregulated proteins in biological process were cellular nitrogen compound metabolic process, biosynthetic process, small molecule metabolic process, cellular protein modification process, and DNA metabolic process. The most abundant GO terms for upregulated proteins under cellular component were intracellular, followed by protein complex, cytoplasm, ribosome and nucleus. Protein complex, nucleus, chromosome, cytoplasm and intracellular were the five top terms for downregulated proteins.

With regard to GO enrichment for upregulated proteins in T vs. C, ion binding, biosynthetic process, extracellular region were the most enriched terms in molecular function, biological process and cellular component respectively. For downregulated proteins, ion binding, structural constituent of ribosome, RNA binding were the top three terms under molecular function and translation, cellular nitrogen compound metabolic process, transport were the most three enriched terms under biological process. The top five terms within cellular component included intracellular, cytoplasm, protein complex, ribosome, nucleus (Figure 4B).

Among molecular function terms in C vs. O, ion binding, oxidoreductase activity, ligase activity, kinase activity and DNA binding were the mostly prevalent terms for upregulated proteins. The most five prominent terms for downregulated proteins were ion binding, oxidoreductase activity, kinase activity, peptidase activity, and lyase activity. Biosynthetic process and small molecule metabolic process occupied top one position in biological process for upregulated proteins and downregulated proteins respectively. The mostly enrichment terms within cellular component for upregulated proteins or downregulated proteins were cytoplasm and intracellular respectively (Figure 4C).

### KEGG pathway analysis of DEPs

**Figure 5:**
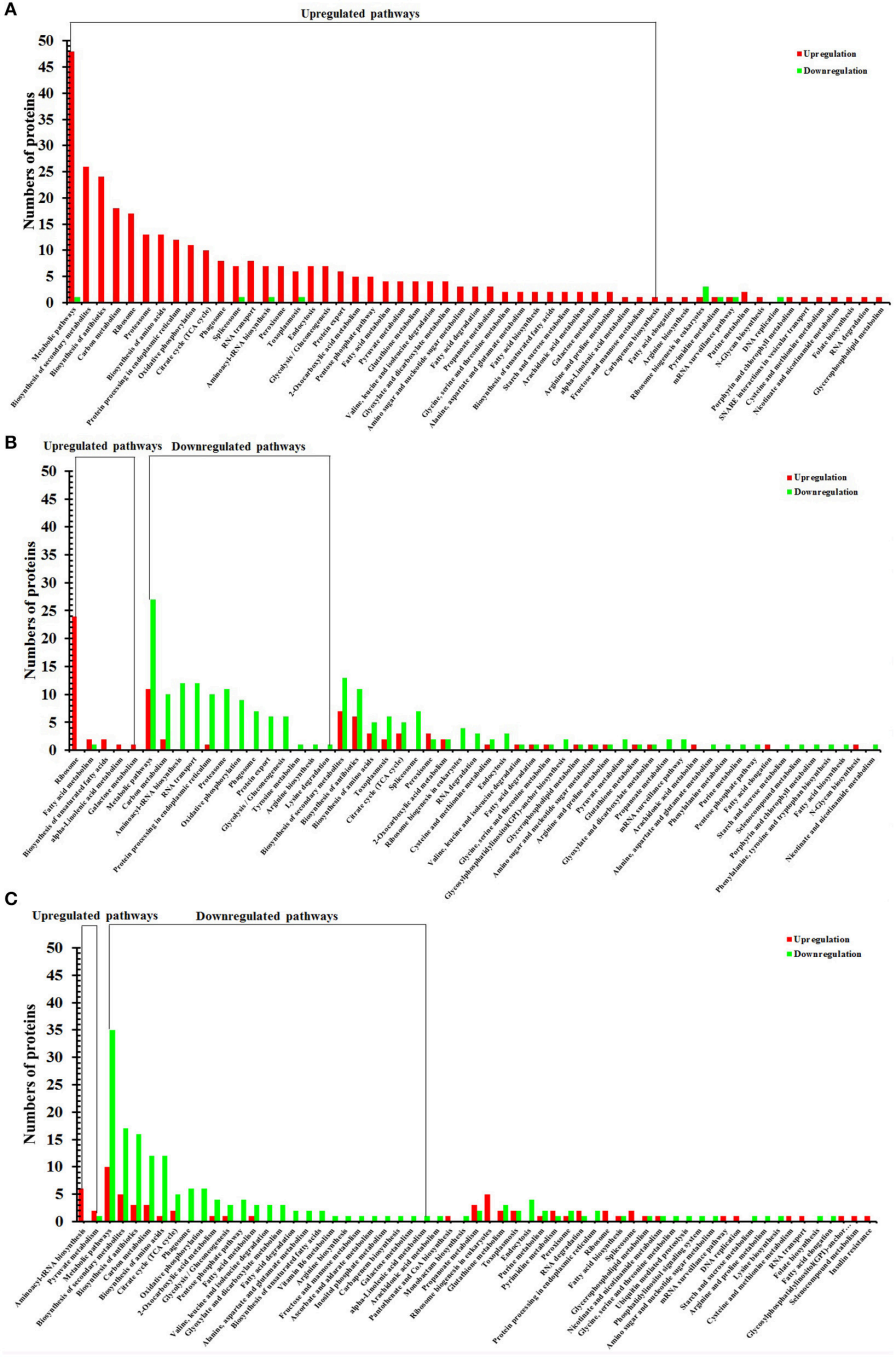
KEGG pathway analysis of DEPs of O vs. T, T vs. C and C vs. O. The x-axis represents different KEGG pathways. The y-axis represents the number of proteins. **(A–C)** represent KEGG pathway analysis of DEPs of O vs. T, T vs. C and C vs. O, respectively. O, oocysts; T, tachyzoites; C, bradyzoites-containing cysts.

To identify the biological pathways operating during *T. gondii* development, we mapped the DEPs in each group to reference pathways contained in the KEGG pathway database. Among DEPs identified in each group, 401 DEPs, 654 DEPs, 536 DEPs had a KEGG Orthology (KO) ID and could be mapped to 55 pathways, 55 pathways, 61 pathways in O vs. T, T vs. C, and C vs. O, respectively. Of the 55 enriched pathways in group O vs. T, 42 pathways were upregulated and no pathway was downregulated. The top 5 enriched upregulated pathways were metabolic pathways, biosynthesis of secondary metabolites, biosynthesis of antibiotics, carbon metabolism, and ribosome (Figure 5A).

There were 5 upregulated pathways and 13 downregulated pathways in T vs. C. Ribosome, fatty acid metabolism, biosynthesis of unsaturated fatty acids, alpha-Linolenic acid metabolism, galactose metabolism were upregulated pathways and the top 5 enriched downregulated pathways included metabolic pathways, carbon metabolism, aminoacyl-tRNA biosynthesis, RNA transport, and protein processing in endoplasmic reticulum (Figure 5B).

In C vs. O, 2 and 25 pathways were up- and down-regulated, respectively. The up-regulated pathways included aminoacyl-tRNA biosynthesis, pyruvate metabolism and the five most enriched downregulated pathways were metabolic pathways, biosynthesis of secondary metabolites, biosynthesis of antibiotics, carbon metabolism, and biosynthesis of amino acids (Figure 5C).

### Analysis of virulence factor expression

**Figure 6:**
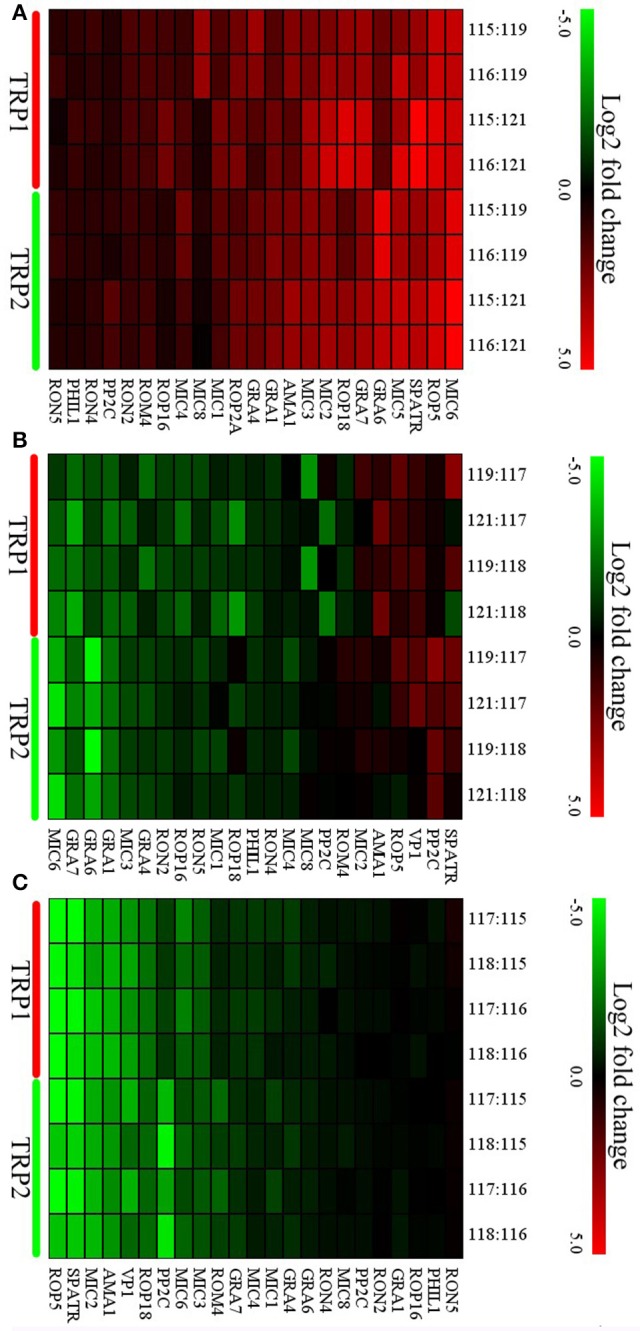
Expression profiles of differentially expressed (DE) virulence factors of O vs. T, T vs. C, and C vs. O. Expression values were log_2_-transformed and the expression levels were annotated with a gradient color scheme. The horizontal axis represents the logogram of virulence factors. The TRP1 and TRP2 in y-axis means biological replicate 1 and 2. **(A–C)** represent expression profiles of DE virulence factors for O vs. T, T vs. C, and C vs. O, respectively. O, oocysts; T, tachyzoites; C, bradyzoites-containing cysts.

To study the expression pattern of virulence factors across the life-cycle stages of *T. gondii*, the expression levels of 23 virulence factors, which simultaneously appear in all groups were analyzed. None of these virulence factors were downregulated and 20 factors were upregulated in oocyst compared with tachyzoite except RON4, PHIL1, and RON5 (Figure 6A). In T vs. C, while five virulence factors (SPATR, PP2C, VP1, ROP5, AMA1) were upregulated in tachyzoite vs. cyst, the number of downregulated virulence factors was 10 (RON5, ROP16, RON2, GRA4, MIC3, ROP2A, GRA1, GRA6, GRA7, MIC6) (Figure 6B). The outcome of comparison between C and O was similar to O vs. T. No virulence factor exhibited higher expression level in cyst and 11 virulence factors were upregulated in the oocysts (GRA7, ROM4, MIC3, MIC6, PP2C, ROP18, VP1, AMA1, MIC2, SPATR, ROP5) (Figure 6C).

### Expression pattern of ribosomal proteins

The differences of ribosomal protein expression among the different life-cycle stages showed upregulation of 33 and 46 ribosomal proteins in oocyst and in cyst, compared to tachyzoite. Six ribosomal proteins had higher expression level in cysts compared with oocyst (Table 1).

**Table 1:** Expression analysis of *Toxoplasma gondii* ribosomal proteins (TgRP).

**TgRP**	**O vs. T**	**T vs. C**	**C vs. O**	**TgRP**	**O vs. T**	**T vs. C**	**C vs. O**
S2	—	—	—	L10A	↑	↓	—
S3	↑	↓	—	L11	—	—	—
S3A	—	↓	—	L12	↑	↓	—
S4	↑	↓	—	L13	↑	↓	—
S5	↑	↓	—	L13A	—	↓	—
S6	↑	↓	—	L14	—	—	—
S7	↑	↓	—	L15	↑	↓	—
S8	↑		—	L17	—	↓	—
S9	↑	↓	—	L18	—	—	—
S10	↑	—	—	L18A	—	—	—
S11	—	↓	↑	L19	↑	↓	—
S12	—	—	—	L21	—	↓	—
S13	↑	↓	—	L22	—	—	—
S14	↑	—	—	L23	↑	—	—
S15	↑	↓	—	L23A	—	↓	—
S15A	↑	↓	—	L24	↑	↓	—
S16	↑	↓	—	L26	↑	↓	—
S17	↑	↓	—	L27	—	↓	—
S18	—	—	—	L27A	—	—	—
S19	—	↓	—	L28	—	—	—
S20	—	—	—	L29	—	—	—
S21	—	↓	↑	L30	—	↓	—
S23	↑	↓	—	L31	—	—	—
S24	—	↓	—	L32	—	↓	—
S25	—	—	—	L34	↑	↓	—
S26	↑	↓	—	L35	—	↓	↑
S27	—	—	—	L35A	—	↓	—
S27A	—	—	—	L36	—	—	—
S28	—	—	—	L37	—	—	—
S29	—	—	—	L37A	—	—	—
S30	—	—	—	L38	↑	↓	—
L3	↑	↓	—	L39	—	—	—
L4	↑	↓	—	L40	—	—	—
L5	↑	↓	—	L41	—	—	—
L6	↑	↓	—	L44	↑	↓	—
L7	↑	↓	↑	SA	—	↓	—
L7A	—	—	↑	P0	—	↓	—
L8	↑	↓	↑	P1	—	—	—
L9	—	↓	—	P2	—	—	—
L10	—	—	—				

*↑, upregulated; ↓, downregulated; —, non-significant changes*.

*O, oocysts; T, tachyzoites; C, bradyzoites-containing cysts*.

### String analysis of protein-protein interactions for DEPs

**Figure 7:**
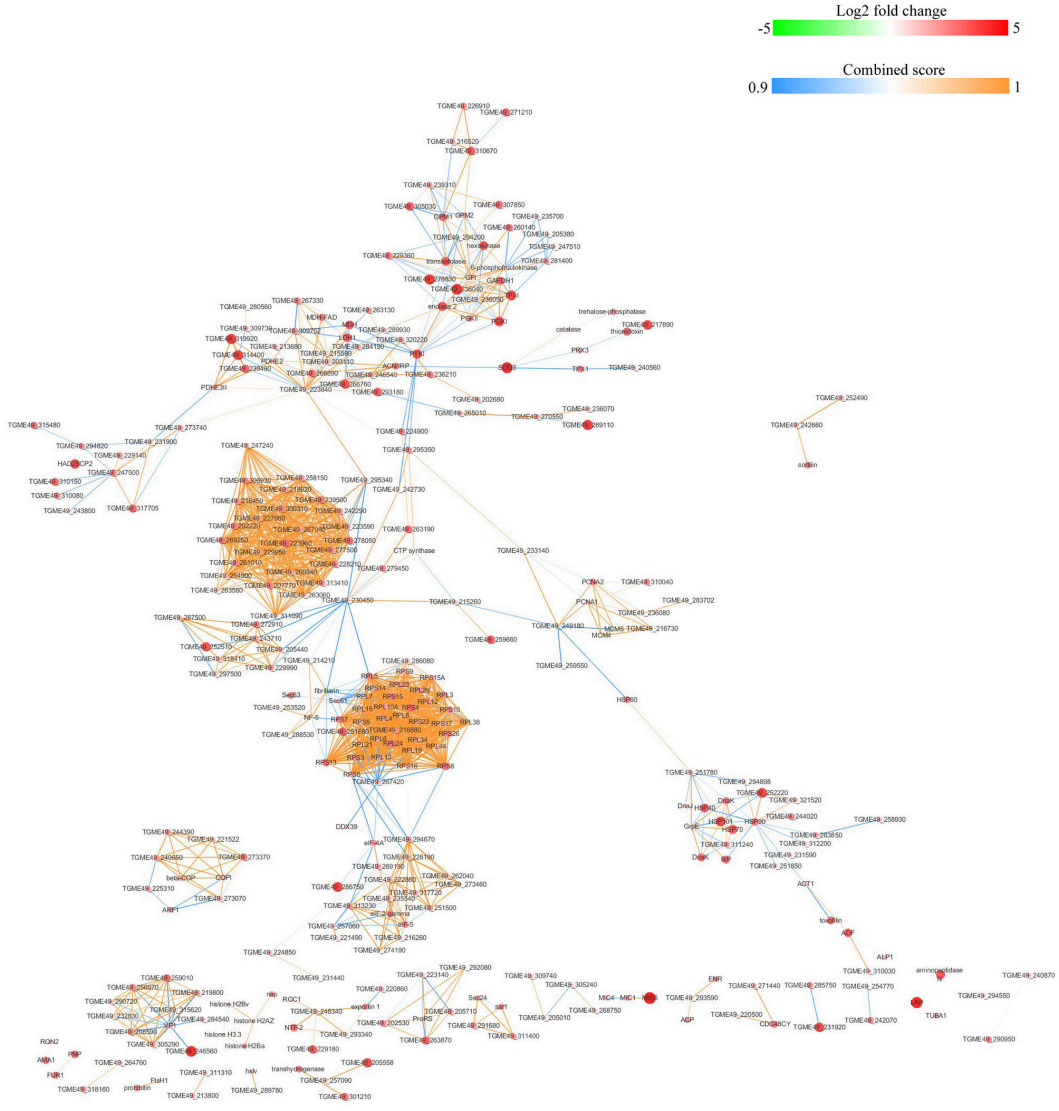
Protein-protein interaction networks of DEPs identified by iTRAQ of oocysts vs. tachyzoites. Proteins were indicated with nodes and interactions between proteins were represented by edges. The node color indicates upregulated protein (red) or downregulated protein (green) and the size of node also presents upregulation (large) or downregulation (small) of DEPs. The combined score were presented with edges colors.

**Figure 8:**
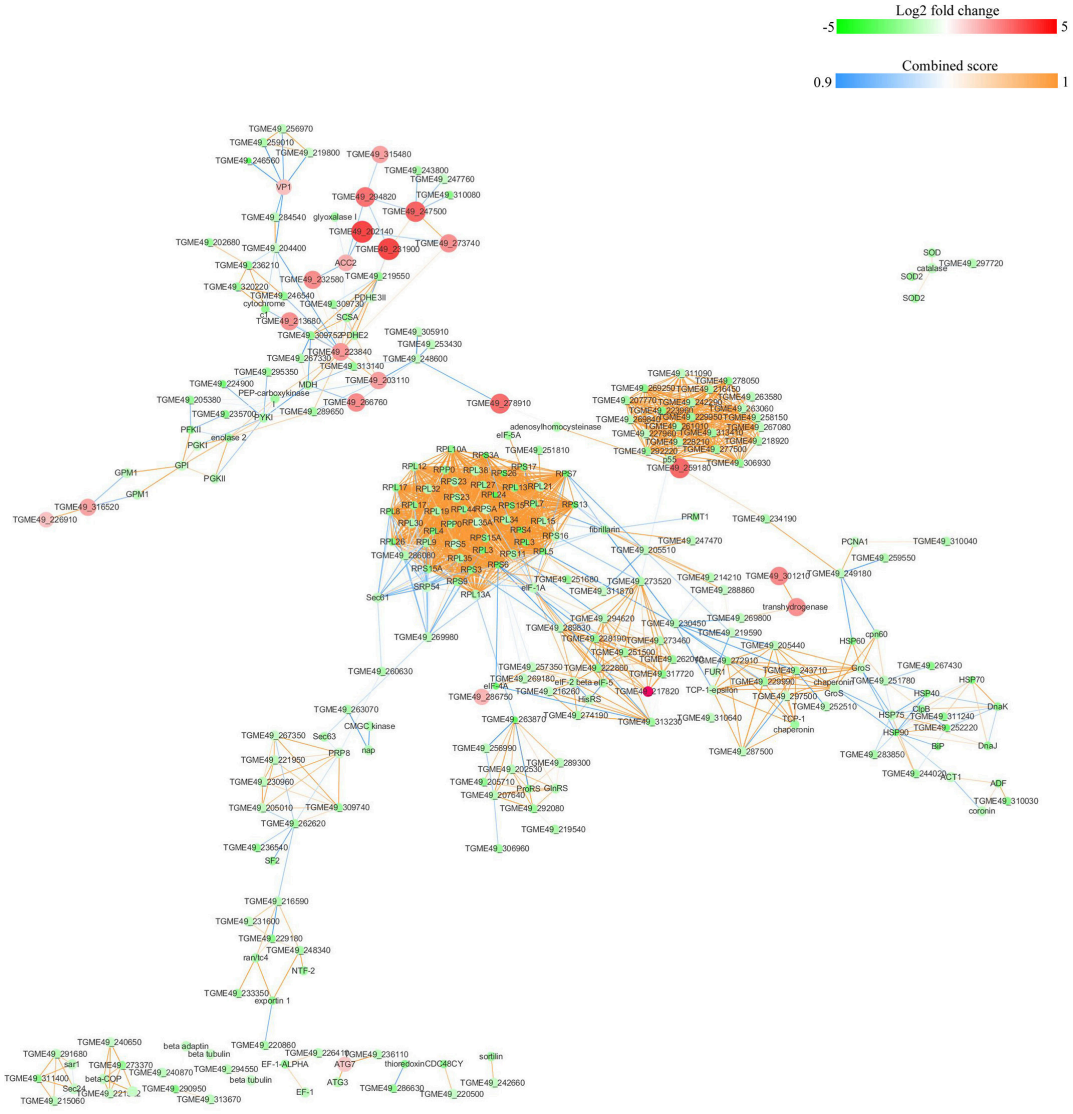
Protein-protein interaction networks of DEPs identified by iTRAQ of tachyzoites vs. bradyzoites-containing cysts. Proteins were indicated with nodes and interactions between proteins were represented by edges. The node color indicates upregulated protein (red) or downregulated protein (green) and the size of node also presents upregulation (large) or downregulation (small) of DEPs. The combined score were presented with edges colors.

**Figure 9:**
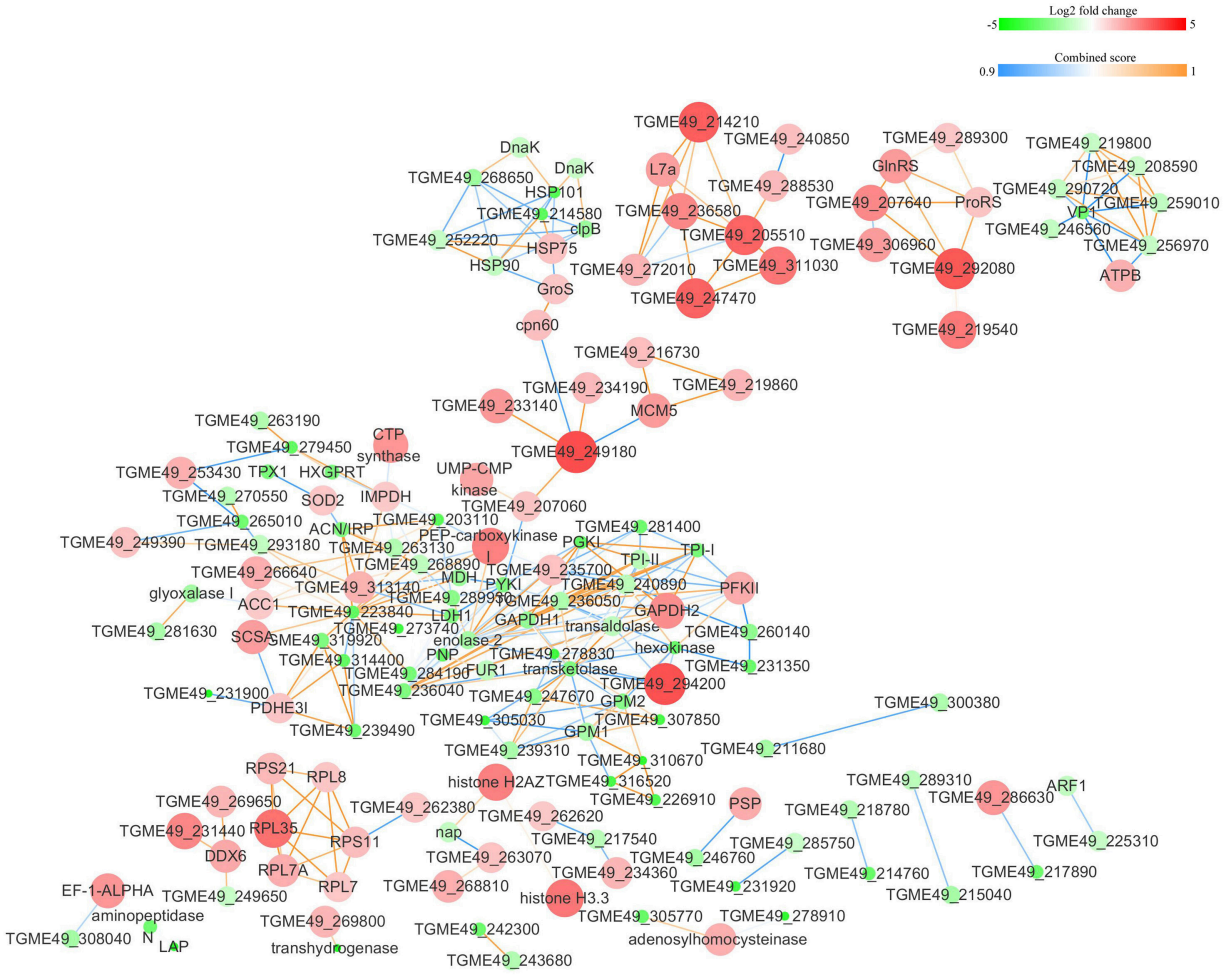
Protein-protein interaction networks of DEPs identified by iTRAQ of bradyzoites-containing cysts vs. oocysts. Proteins were indicated with nodes and interactions between proteins were represented by edges. The node color indicates upregulated protein (red) or downregulated protein (green) and the size of node also presents upregulation (large) or downregulation (small) of DEPs. The combined score were presented with edges colors.

The protein-protein interactions (PPI) whose combined score was >0.9 were used to build network using Cytoscape tool in each group. As shown in Figures 7, 8, there were 310 nodes and 1501 edges in the PPI network of O vs. T, and 273 nodes and 1769 edges in the PPI network of T vs. C. The PPI network of C vs. O contained 152 nodes and 308 edges (Figure 9).

## Discussion

In order to understand the functional differences across the different life-cycle stages of *T. gondii*, it is important to identify, quantify and compare the protein repertoire of each of these life cycle forms. In the context of host-pathogen interaction, such insights have the potential to reveal new mechanistic determinants of *T. gondii* infectivity and adaptation. In this study, we applied iTRAQ labeling coupled with LC-MS/MS approach to profile and compare the proteome of *T. gondii* oocysts, tachyzoites and bradyzoites-containing cysts. A total of 6285 proteins were identified in the three developmental stages of *T. gondii*. The comparative analysis identified significant differences in the level of expressed proteins when comparing oocysts to tachyzoites (875 proteins), tachyzoites to cysts (656 proteins) and cysts to oocysts (538 proteins). Based on GO and KEGG analyses, ribosomal proteins and proteins involved in macromolecule metabolism were the predominant groups.

A number of virulence-related factors and ribosomal proteins exhibited distinct expression patterns across the life-cycle stages. Some virulence factors can be upregulated during the sporulation of oocysts (Wu et al., 2006). In our study, a number of proteins with a virulence function have been identified. Compared with oocysts, none of virulence factors was significantly overexpressed in tachyzoite and cyst stages. By contrast, 23 and 11 virulence factors were upregulated in oocyst compared with tachyzoites and cysts, respectively. This indicates that oocysts expressed more virulence factors compared to other stages of the life-cycle of *T. gondii*. Interestingly, the number of upregulated virulence factors was twice the number of downregulated factors in cyst compared with tachyzoite. These findings seem consistent with previous data, which reported that genes coding for β-oxidation enzymes are not expressed in tachyzoites, but may be active in oocysts (Possenti et al., 2013). Additionally, the dense granule protein GRA1 (a specific protein that is known to be expressed at the transformation from tachyzoites to bradyzoites) was found down-regulated in tachyzoites compared to cysts in our study. This finding is also consistent with pervious work, which reported a progressive suppression in the expression of this protein during the formation of bradyzoites-containing tissue cysts (Cleary et al., 2002). GRA1 protein has been shown to be up-regulated in sporulated oocysts as well (Tilley et al., 1997; Possenti et al., 2013). The biological and clinical relevance of these differences remain to be investigated using functional assays.

Previous studies have shown that certain proteins might be functional in certain life-cycle stages. For example, while the homoserine kinase has been observed in the proteome of oocysts (Possenti et al., 2013), genes encoding for homoserine kinase (TGME49_216640) and other enzymes in the threonine biosynthesis pathway, such as aspartokinase (TGME49_227090), aspartate-semialdehyde dehydrogenase (TGME49_205420), and threonine synthase (TGME49_220840), are known to be expressed at low levels in the tachyzoite stage. In agreement with these studies our results based on comparing oocysts to tachyzoites' DEPs showed kinase activity amongst the top five enriched GO terms for differentially regulated proteins within molecular function. Our study also identified proteins with greater abundance that were involved in biological process, such as cellular nitrogen compound metabolic process, biosynthetic process, small molecule metabolic process, cellular protein modification process, and DNA metabolic process in the tachyzoites compared to oocysts. This finding supports the higher bioenergetics and metabolic need of *T. gondii* replicative forms (tachyzoites), which rely heavily on glucose uptake and glycolysis for generation of ATP and other intermediates required for energy generation and replication (Al-Anouti et al., 2004).

The ribosome is the factory for protein synthesis that sustains the development of biological organisms. It is assembled from both rRNA and ribosomal proteins (Meyuhas, 2000). The synthesis and expression of ribosomal proteins is higher in rapidly dividing cells and are regulated when the cell is under stress or is stimulated by growth factors (Pearson and Haber, 1980; Ju and Warner, 1994; Zhang et al., 2013). Various coccidian protozoan organisms are known to regulate the transcription of ribosome biosynthesis in order to adapt to changes that accompany stage transitions during their developmental life-cycles (Meyuhas, 2000; Cleary et al., 2002). *Toxoplasma gondii* contains 79 different ribosomal proteins, whose encoding genes are randomly distributed across the *T. gondii* genome (Tilley et al., 1997; Van Poppel et al., 2006). Seventy-one of the 79 ribosomal protein loci are distributed as single loci across the genome, but 8 loci are unique in that they are paired at four locations in a head to head arrangement. In our study, oocysts and cysts showed the highest expression of ribosomal proteins compared to tachyzoites, indicating that these two stages have more metabolic flexibility, which may contribute to their ability to adapt to stress conditions. Oocysts are the environmental infective forms of *T. gondii*, and can survive for extensive periods of time outside the host (Torrey and Yolken, 2013). A previous study indicated that there are stage-associated differences in the expression of *T. gondii* ribosomal proteins (Schaap et al., 2005). Intriguingly, Hutson et al. showed via the generation of Δ*rps13* strain-deficient in ribosomal protein S13 (RPS13) that disruption of ribosomal proteins can lead to arrest of *T. gondii* cell cycle, resulting in dormant parasites which can persists for several months *in vitro*, but without forming mature tissue cysts (Hutson et al., 2010). The same authors also demonstrated that immunization using the attenuated parasite Δ*rps13* strain can protect mice against subsequent challenge with wildtype *T. gondii*. However, it remains to be elucidated which of the differentially expressed ribosomal proteins identified in our study are essential for *T. gondii* ribosomes to function properly in the respective stages of the life-cycle.

## Conclusion

In the present study, we used iTRAQ-based LC-MS/MS technique to compare the abundance of proteins in three life-cycle forms of *T. gondiii* (oocyst, tachyzoite and bradyzoites-containing cyst). Our results revealed differences in the protein expression among the three life-cycle stages of this parasite, providing new insight into the adaptation mechanisms of these life-cycle forms to their habitats. These results lay the foundation for the understanding of the developmental biology of *T. gondii*, and should facilitate the discovery of novel therapeutic targets and the development of novel stage-specific diagnostic assays to augment available prevention measures to control toxoplasmosis.

## Author contributions

XZ and DZ conceived and designed the experiments. ZW, CZ, and SH performed the experiments. DZ contributed reagents/materials/analysis tools. ZW analyzed the data and wrote the paper. ZW, HE and XZ critically revised the manuscript. All authors read and approved the final version of the manuscript.

### Conflict of interest statement

The authors declare that the research was conducted in the absence of any commercial or financial relationships that could be construed as a potential conflict of interest.

## Supplementary material

### Availability of data and material

The datasets supporting the conclusions of this article are included within the article and its additional files. The mass spectrometry proteomics data of this article are available in the ProteomeXchange Consortium and the data set identifier is PXD003765.

Figure S1 The chromatogram of SCX fractionation and LC- MS/MS analysis.

Table S1 List of identified proteins between oocysts and tachyzoites after filtration (CV ≤ 0.5).

Table S2 List of identified proteins between tachyzoites and cysts after filtration (CV ≤ 0.5).

Table S3 List of identified proteins between cysts and oocysts after filtration (CV ≤ 0.5).

Table S4 List of identified proteins between oocysts and tachyzoites using 99% confidence level (CV ≤ 0.5).

Table S5 List of identified proteins between tachyzoites and cysts using 99% confidence level (CV ≤ 0.5).

Table S6 List of identified proteins between cysts and oocysts using 99% confidence level (CV ≤ 0.5).

Table S7 List of differentially expressed proteins (DEPs) between oocysts and tachyzoites (|log2 fold change|>1 and *P* < 0.05).

Table S8 List of differentially expressed proteins (DEPs) between tachyzoites and cysts (|log2 fold change|>1 and *P* < 0.05).

Table S9 List of differentially expressed proteins (DEPs) between cysts and oocysts (|log2 fold change|>1 and *P* < 0.05).

## Abbreviations

SPF: specific pathogen free
PBS: phosphate buffered saline
DEPs: differentially expressed proteins
2-DE: 2-dimensional electrophoresis
iTRAQ: isobaric tags for relative and absolute quantification
MALDI-TOF-MS: matrix-assisted laser-desorption/ionization time of flight mass spectrometry
2D-DIGE: two-dimensional fluorescence difference gel electrophoresis
FDR: false discovery rate
GO: Gene Ontology
KEGG: Kyoto Encyclopedia of Genes and Genomes
PPI: Protein-protein interactions
CV: Coefficient of variation.
