# Supplementary figures and images for: Proteomic Differences between Developmental Stages of Toxoplasma gondii Revealed by iTRAQ-Based Quantitative Proteomics
Source: Front Microbiol. 2017 Jun 2;8:985. doi: 10.3389/fmicb.2017.00985 (PMC5454076; doi:10.3389/fmicb.2017.00985)

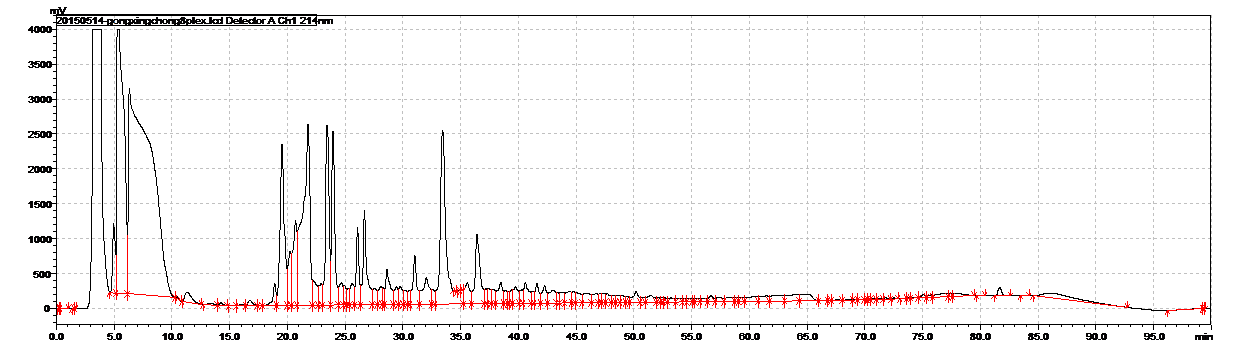

Supplement: Figure S1 — The chromatogram of SCX fractionation and LC- MS/MS analysis. [file Image1.PNG]
